# The Completion of Postoperative Radiotherapy After Breast-Conserving Surgery in a Patient With Recurrent Erythema Multiforme: A Case Report

**DOI:** 10.7759/cureus.61760

**Published:** 2024-06-05

**Authors:** Satoshi Teramura, Yojiro Ishikawa, Kengo Ito, Takayuki Yamada

**Affiliations:** 1 Radiology, Tohoku Medical and Pharmaceutical University, Sendai, JPN

**Keywords:** radiation therapy, breast cancer, erythema multiforme, dermatitis, radiation

## Abstract

Radiotherapy (RT) can induce dermatitis and exacerbate a patient's preexisting skin conditions. We present a case of RT in a 61-year-old Japanese woman with a history of erythema multiforme (EM). She was diagnosed with a nodule on her right breast during therapy for EM. EM was noticed on the anterior chest and upper and lower extremities. RT was initially postponed due to exacerbation of EM before postoperative RT for right breast cancer. However, considering that EM tends to recur every one to two months, RT was commenced during a period of less active dermatitis, and a total dose of 50 Gy of conventional irradiation was successfully administered. One year after RT, there was no EM recurrence, dermatitis development, obvious late effects, or radiation pneumonitis. Our experience suggests that RT can be administered relatively safely to patients with recurrent EM but should be administered with caution.

## Introduction

Patients undergoing radiotherapy (RT) may experience dermatitis or recall phenomena in the RT field [1]. Radiodermatitis is a common complication in patients receiving RT, with skin reactions typically confined to the irradiated field and being dose-dependent [2]. Recall phenomena usually manifest within two months after completion of RT and within one to 14 days after initiation of chemotherapy [3]. This phenomenon involves the relapse of dermatitis following chemotherapy or other drug administration and has also been reported after coronavirus vaccination [4]. Ionizing radiation can induce hypersensitivity in certain diseases, often due to genetic predisposition. Ataxia telangiectasia, Seckel syndrome, Nijmegen fracture syndrome, and Fanconi anemia are significant conditions that may contraindicate RT [3]. RT in patients with dermatitis or skin diseases can also exacerbate these conditions. However, there is currently no well-established policy regarding RT for patients with preexisting skin diseases. Here, we present a case of postoperative breast-conserving irradiation in a patient with recurrent EM prior to irradiation.

## Case presentation

**Figure 1:**
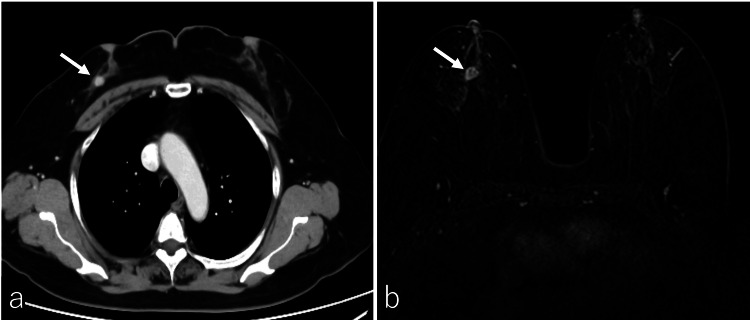
Computed Tomography and Magnetic Resonance Imaging Computed tomography (CT) revealed a 13 mm nodule in the lower lateral aspect of the right breast with no apparent metastatic lesions (a, arrow). Magnetic resonance imaging (MRI) showed a 12 mm nodule in the right breast, consistent with the CT findings (b, arrow).

A 61-year-old Japanese woman developed skin rashes on her extremities. The rash appeared as erythematous target-like lesions with a purple to gray color at the center and an overall red tone, initially appearing on the palms, soles, arms, legs, and face, before spreading to the rest of the body without stomatitis. Biochemical, bacterial, and viral screening tests did not reveal an obvious cause, but a diagnosis of erythema multiforme (EM) was made. The patient's condition did not involve severe exudative erythema or mucosal sores. Steroids were not prescribed, and regular use of antihistamines was unnecessary because the itching was not severe. After three months, imaging studies were performed for a mass in the right breast that was found incidentally during EM treatment. Computed tomography (CT) revealed a 13 mm nodule in the lower lateral side of the right breast with no evident metastatic lesions (Figure 1a). Magnetic resonance imaging (MRI) showed a nodule of approximately 12 mm in the right breast, consistent with the findings on CT (Figure 1b).

**Figure 2:**
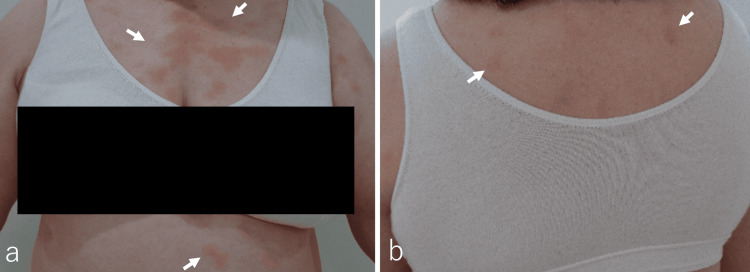
Gross Findings Before Radiation Therapy Rashes manifested as erythematous target-like lesions with a purple to gray center and overall red coloration on the anterior chest wall (a, arrows) and back (b, arrows).

**Figure 3:**
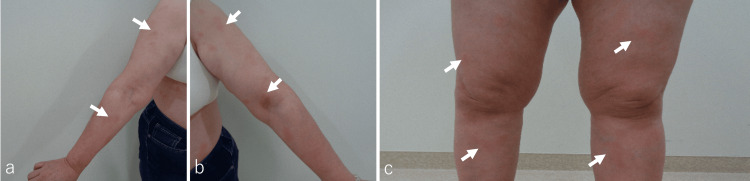
Gross Findings Before Radiation Therapy Rashes presented as erythematous target-like lesions with a purple to gray center and overall red coloration on the palms, soles, arms (a and b, arrows), and legs (c, arrows), before spreading to the remainder of the body without stomatitis.

The diagnosis of invasive ductal carcinoma of the breast was confirmed through core needle biopsy, resulting in a final diagnosis of cT1c, cN0, cM0, ER(+), PgR(+), HER2(-), Ki-67 (10%), and stageⅠA. A partial right mastectomy was performed. The postoperative diagnosis was pT1a, pN0, cM0, tumor size 10 mm, surgical margin (-), lymphatic invasion (-), and vascular invasion (-). One month after surgery, the wound was stable, and the patient was scheduled for postoperative RT. At the time of consultation before a treatment planning CT scan, the patient's performance status was 1 on the Eastern Cooperative Oncology Group scale, but there was a flare-up of EM on the anterior chest, back (Figures 2a, 2b), upper extremities, and lower extremities (Figures 3a, 3b), including the planned radiation field. Due to the exacerbation of EM, the patient's treatment plan was postponed. At a follow-up visit one month later, the EM had improved.

**Figure 4:**
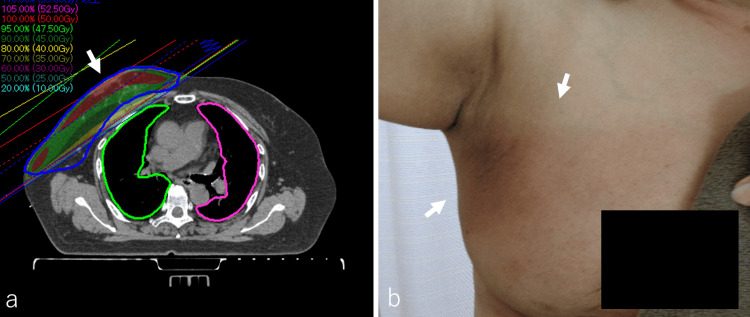
Radiation Field and Dose Distribution of Radiation Therapy, and Examination Findings Post-radiation Therapy The radiotherapy (RT) plan was generated using the RayStation treatment planning system (RaySearch Laboratories AB, Stockholm, Sweden). The patient consented to receive 50 Gy in 25 fractions over a period of five weeks, administered via a 6-megavoltage device utilizing a multileaf collimator. The clinical target volume (CTV) was the right breast (a, arrow), and the planned target volume (PTV) encompassed the CTV plus a 1.0 cm margin. Although acute radiation dermatitis occurred, they did not exceed grade 2 severity. The patient completed the RT without recurrence of erythema multiforme (b, arrows).

The disease course indicated that the interval between EM episodes was approximately one to two months, and subsequent flare-ups were relatively mild compared to the initial occurrence. After another month, the erythema had further improved, and the treatment plan was devised to complete RT before the next relapse. Following an explanation of the risks and benefits of RT to the right breast in the presence of recurrent EM, the patient consented to receive 50 Gy in 25 fractions over a period of five weeks, delivered by a 6-megavoltage device via a multileaf collimator. The clinical target volume (CTV) was the right breast, and the planned target volume (PTV) included the CTV plus a 1.0 cm margin. Acute complications occurred, but there was no grade 2 or higher acute complication (Figure 4a, 4b).

**Figure 5:**
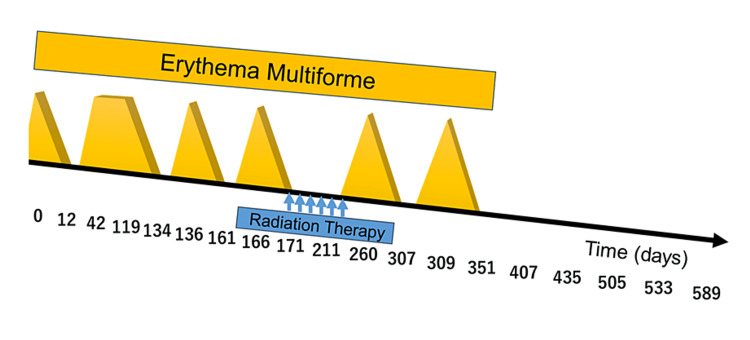
Timeline of Chronological Changes in Erythema Multiforme and Timing of Irradiation There had been recurrent exacerbations and remissions of erythema multiforme since the patient's initial presentation. Radiotherapy was completed during a period of remission.

The patient completed the RT without recurrence of EM. While erythema appeared completely after irradiation, all instances were manageable with conservative treatment. One year post-RT, there was no exacerbation of EM, no dermatitis, no obvious late effects, and no radiation pneumonitis. A timeline depicting the pattern of recurrent EM onset and the timing of RT administration is presented in Figure 5.

## Discussion

Radiation-induced skin reactions are classified on the basis of severity into EM, Stevens-Johnson syndrome (SJS), and toxic epidermal necrolysis (TENS) [5]. These reactions can range from mild to severe, extending to the irradiated field, and in severe cases, involving the eyes and mucosa. They may be triggered by infectious agents such as viruses and bacteria, medications, radiation, collagen diseases, and malignancies [6]. EM is commonly triggered by infectious diseases, while SJS and TENS are often caused by medications such as antibiotics, anticonvulsants, and nonsteroidal anti-inflammatory drugs [6]. Although our patient did not use antibiotics or anticonvulsants, it was anticipated that RT had the potential to exacerbate EM. As far as we can determine, no similar cases have been reported to date. However, various theories regarding the mechanism by which RT contributes to the development of SJS and TENS have been proposed. One theory is the involvement of cytochrome P450 in metabolizing drugs such as phenytoin, phenobarbital, carbamazepine, and sulfonamides into toxic reactive metabolites, which can trigger secondary immunological reactions [7-9]. There have been reported cases of EM, SJS, and TENS occurring solely after RT, suggesting that radiation itself may exacerbate these skin diseases [10-12].

In cases in which EM has already developed, RT may exacerbate the condition and pose a risk of inducing SJS and TENS [13]. Radiodermatitis is common after local RT for various cancers and may necessitate treatment interruption in severe cases. Recent research suggests that intense inflammatory responses disrupting skin barrier function and cytokine release may exacerbate radiation-induced dermatitis [14]. Fortunately, in this case, treatment was completed successfully. Recurrence of EM is known, and healing may take time. According to previous reports and guidelines, RT should be started within 4, 12, or 20 weeks after breast-conserving surgery, but it should generally be started within 20 weeks. However, in cases of recurrent EM, the initiation of RT may be delayed [15-17]. Caution should be exercised when considering RT for patients with other diseases or other complications that may be negatively impacted by RT [18]. Depending on the type of concomitant skin condition, treatment completion after dermatitis resolution may be reasonable. Recognizing the potential to irradiate during a relatively low-risk period is important, as demonstrated in this case report, where monitoring for skin conditions before treatment initiation is advisable. Our patient had a history of EM, which influenced the timing of initiating postoperative irradiation. Effective communication with the surgeon before surgery is essential. In certain situations, the possibility of forgoing postoperative RT should be taken into account.

## Conclusions

This case highlights breast-conserving irradiation in a patient with recurrent EM, suggesting the possibility of irradiation use while monitoring dermatitis patterns. Omitting radiation therapy could also be considered, depending on the severity of EM.

RT may be difficult to administer if there is a skin disease at the site of RT. RT can induce dermatitis and exacerbate a patient's preexisting skin disease. Irradiation of patients with preexisting skin disease should be carefully considered. This is the report of a rare case of safe breast-conserving irradiation in a patient with EM, and it may be useful for clinicians encountering similar cases.
